# Heat, menstruation, and mental health in adolescence and young adulthood: Understudied in climate-health research

**DOI:** 10.1016/j.dialog.2026.100344

**Published:** 2026-08-19

**Authors:** C. Brimicombe, J.D. Runkle, B. Mouchtouri, C. Cattle, C. Part, F. Kalala, H. Marshall, I. Voulgaridi, K. Wan, M. Tembo, S. Hajat, V. Simms, P. Banati

**Affiliations:** a Department of Social Policy Intervention, University of Oxford, Oxford, UK; b North Carolina Institute for Climate Studies, North Carolina State University, United States of America; c Laboratory of Hygiene and Epidemiology, Faculty of Medicine, University of Thessaly, 41222 Larissa, Greece; d Irise International, Registered Charity No. 1157722, UK; e Department of Public Health, Environments and Society, London School of Hygiene and Tropical Medicine, UK; f Health Protection Research Unit in Climate Change and Health Security, The National Institute for Health and Social Care Research, UK; g Department of Immunology and Histocompatibility, Faculty of Medicine, University of Thessaly, 41500 Larissa, Greece; h Thru Zim, 8 Ross, Harare, Zimbabwe; i Department of Global Health and Development, Faculty of Public Health and Policy, London School of Hygiene and Tropical Medicine, London, UK; j Department of Infectious Disease Epidemiology, London School of Hygiene and Tropical Medicine, Geneva, Switzerland; k Department of Maternal, Newborn, Child and Adolescent Health and Ageing, World Health Organization, United States of America

**Keywords:** Mental health, Menstrual health, Heat, Extreme heat, Extreme temperature, Adolescent girls, Young women, Women's health, Climate and health, Climate change, Biomarkers, Life course

## Abstract

Ambient heat is an escalating climate-related hazard, yet its implications for menstruation and adolescent and young adult mental health remain understudied. Menstruating individuals face unique physiological and psychosocial challenges that may be intensified in the presence of heat stress. These challenges can be further shaped by stigma, period poverty, and developmental vulnerability, with potential implications for depression, anxiety, and psychological wellbeing. Heat exposure may disrupt thermoregulation, exacerbate menstrual symptoms, and heighten psychological stress, but the combined effects of these processes remain poorly understood. In this critical narrative review, we: draw together evidence from multiple disciplines on biological, psychological, and structural pathways linking heat exposure, menstruation, and mental health. We identify important research gaps and propose a conceptual framework and gender-responsive research agenda to inform equitable climate-resilient health strategies.

## 1 Introduction

Adolescence and young adulthood are critical life course transitions when vulnerability to mental health disorders first emerge. Globally, an estimated 50% of lifetime mental health conditions begin by age 14 and 75% by age 24, highlighting the importance of early intervention during these stages [1], [2]. Although depressive disorders are relatively uncommon in children, this condition becomes nearly twice as prevalent in females compared to males after puberty and continues to rise through late adolescence and early adulthood [3], [4]. During this transitional period into and out of puberty, young people experience profound hormonal shifts, neurodevelopmental reorganization, and evolving social roles that heighten susceptibility to environmental and psychosocial stressors [5], [6].

As one of the defining health challenges of the 21st century, climate change is driving increases in the frequency, duration, and intensity of heatwaves worldwide [7], [8]. Extreme heat not only strains human thermoregulation and healthcare systems but also deepens existing social and gender-based health disparities [9]. For young people already managing pre-existing mental health vulnerabilities, such as anxiety, depression, or trauma, the destabilizing effects of environmental stressors like heatwaves may be especially pronounced [12]. Thermal stress can disrupt sleep, impair emotion regulation, and diminish cognitive function, all of which are essential to mood regulation during adolescence [13], [14]. Beyond physiological strain, rising temperatures contribute to “eco-anxiety”, the negative emotions of worry, fear, and anxiety related to climate change and environmental degradation, further amplifying distress in young people [15], [16], [17].

Despite these converging risks, one critical dimension remains largely overlooked: menstruation. Experienced by more than half of the world's population, menstruation represents a cyclical biological process with direct ties to thermoregulation, hormonal balance, and emotional health. Yet its intersection with environmental exposures, particularly extreme heat, is under researched [18]. This omission reflects a persistent structural bias in women's health research, which continues to receive disproportionately low levels of funding and attention relative to conditions predominately affecting males [19]. In climate and health research more specifically, the female focus is overwhelmingly on maternal health, with limited attention on other stages of the life course, such as adolescence and young adulthood, periods when menstrual and mental health vulnerabilities first debut and when intervention measures are likely to be most effective [20].

This critical narrative review proposes a conceptual framework using a life-course approach linking extreme heat, menstruation, and mental health in adolescents and young adults, highlighting pathways of vulnerability and resilience across biological, social, and environmental determinants. Throughout this review, we use ‘menstruating individuals’ when referring to biological processes related to menstruation and ‘adolescent girls and young women’ when discussing epidemiologic evidence, social experiences, and gender disparities. This critical narrative aims to draw attention to an under-resourced area to inform policy and practice that integrate mental and menstrual health within climate adaptation planning, strengthening health systems and advancing gender and climate equity globally.

## 2 Method

This study uses a critical narrative review approach to expertly review the literature across the interdisciplinary fields of climate and environmental health, menstrual health, adolescent and young adult mental health, and reproductive health [20]. A critical narrative review was chosen because the objective was to integrate diverse bodies of literature, identify conceptual and empirical gaps, and develop a testable framework for future research.

Literature was identified through searches of major biomedical databases, including PubMed and Google Scholar. The inclusion criteria looked at relevant literature on Mental Health, Menstrual Health, Heat-Health, with a focus on the adolescent and young adult life stages. The review focuses on including only literature from peer-reviewed articles in the English language supplemented by other reviews and thought‑leadership publications where appropriate.

Studies were included if they addressed one or more of the following domains: (1) heat exposure and health; (2) menstrual health or physiology; (3) adolescent or young adult mental health; or (4) psychosocial or social determinants relevant to these topics. Evidence was synthesized narratively to identify recurrent themes, plausible biological and psychosocial pathways, and areas where evidence converged or remained limited.

This review takes a global health perspective to allow for inclusion of studies on heat, mental health and menstrual health from both the Global South and North to be assessed. This is presented as a narrative for before leading to the creation of an overarching conceptual framework.

## 3 Results

### 3.1 Menstruation: a biological and social determinant

#### 3.1.1 Biological dimensions

The menstrual cycle is a biological rhythm directly tied to thermoregulation and endocrine function. During each cycle, basal core body temperature rises approximately 0.3 to 0.5 °C in the luteal phase, a thermogenic effect of progesterone [18]. When environmental heat coincides with this phase, it may intensify an endogenous rise in core body temperature [21], placing additional physiological heat strain and exacerbating menstrual symptoms such as fatigue, irritability, and dysmenorrhea (i.e., menstrual pain) [22].

Despite growing recognition of sex-based differences in heat tolerance, the mechanistic pathways by which thermal stress may interact with hormonal fluctuations remain poorly understood. This knowledge gap is particularly pronounced for adolescents and young adults, whose hormonal systems are still maturing [23]. Emerging evidence suggests that heat exposure may alter endocrine and inflammatory processes, influencing menstrual dysfunction and mood, but definitive models are lacking [24]. We hypothesize that the hormonal fluctuations regulating the menstrual cycle may amplify susceptibility to the destabilizing effects of environmental heat, both physiologically through increased thermal strain and psychologically through altered mood regulation. These effects may be magnified in settings where health resources are limited or menstruation remains stigmatized.

#### 3.1.2 Social dimensions

Menstruation is not merely a biological process, but it is a deep social experience mediated by structural factors, cultural norms, and access to material and informational resources [25]. For adolescents navigating puberty, identity formation, and new social expectations, these social dimensions shape both how menstruation is experienced and how it is managed [5]. Across the globe, gender interacts with poverty, education, and health infrastructure to determine unequal exposure to environmental hazards and unequal capacity for adaptation [26].

We propose that menstruation functions as a social determinant of climate vulnerability, where socio-cultural, structural, and environmental dimensions converge to intensify both physical and emotional strain during periods of extreme heat. Menstrual health is strongly influenced by period poverty, a multidimensional structural condition encompassing not only limited access to affordable menstrual products, but also inadequate water, sanitation and hygiene (WASH) infrastructure, lack of privacy and safe disposal facilities, menstrual stigma, restricted mobility, and barriers to full participation in school, work, and community life. These interconnected challenges have important implications for dignity, educational attainment, school participation, and mental wellbeing [26], [27]. Millions of women and girls worldwide face shame, stigma, and restricted mobility due to inadequate menstrual management resources.

In low-income settings, vulnerability to period poverty is often heightened. In Bangladesh, it is described that period poverty is limited access to menstrual products, sanitation, and education, and shown how these constraints affect nursing students' product choices, disposal practices, and emotional experience [28]. In Zambia, period poverty is framed as a public health problem linked to inadequate product access and unsafe substitutes, with rural learners especially vulnerable and facing weaker knowledge, attitudes, and practices [29]. In Tanzania, it is argued that menstrual health management difficulties are a hidden obstacle to girls' participation in school and academic achievement, helping explain why equal enrollment has not led to equal exam performance [30]. Taken together, it is shown that period poverty is not only a hygiene issue but a structural inequality affecting health, schooling, and dignity.

Period poverty is not confined to low-income settings, although nuances in the lived experience are different across geographic contexts. A recent nationally representative UK survey of 2000 adults revealed that menstrual stigma, discrimination, and embarrassment remain pervasive [31]. Nearly one in four people who menstruate reported difficulty affording period products, increasing to nearly one in three in GenZ, disabled, and racially minoritized respondents [31]. Despite generational progress, embarrassment and societal discomfort persist, demonstrating how stigma continues to mediate social participation, confidence, and wellbeing. During periods of extreme heat, inadequate access to cooling, water, sanitation, privacy, and menstrual products may further complicate menstrual management, increasing physical discomfort, psychological stress, and barriers to participation in daily activities. These intersecting social and environmental stressors highlight why menstrual health should be considered within climate adaptation and resilience planning.

#### 3.1.3 Psycho-social dimensions

These burdens are further heightened during periods of extreme heat and other climate hazards, where resource disruptions to water and sanitation infrastructure, and displacement can constrain access to menstrual products, amplifying shame and discomfort, and emotional distress [32], [33], [34]. For adolescents and young adults, these experiences may reinforce psychosocial stress at a developmental stage already characterized by heightened sensitivity to emotional distress.

Psychosocial stress contributes to menstrual dysfunction through disruption of the hypothalamic–pituitary–gonadal axis, leading to irregularities, dysmenorrhea (painful menstruation), or amenorrhea (absence of menstruation) [35], [36], [37]. Conversely, menstrual dysfunction may also be a cause of psychological stress, forming a bidirectional feedback loop [38], [39]. Although both extreme heat and menstrual irregularities have been independently associated with increased psychological stress, their combined effects and the potential mediating role of social determinants such as period poverty, remain largely unexplored.

Exploring this gap is crucial for developing gender-responsive adaptation strategies that recognize menstruation as both a biological and socially embedded process, shaped by the broader constellation of climate-related stressors, such as heat exposure, food or water insecurity, and infectious disease. The absence of empirical studies examining how heat may interact with menstrual health to influence anxiety, depression, or other psychiatric outcomes in adolescent or young adults highlights an important evidence gap. Exploring this gap could help reframe menstrual health as a climate-sensitive public health priority within climate adaptation and resilience planning.

### 3.2 Mental health: adolescence and young adulthood as critical periods

Adolescence and young adulthood represent sensitive developmental periods when the majority of mental health disorders first emerge. Rapid biological, cognitive, and social changes, coupled with pubertal maturation, evolving identity, and growing social pressures, make adolescents and young adulthood particularly sensitive to environmental stressors that can alter brain development, emotion regulation, and long-term mental health trajectories [5], [34]. These vulnerabilities may be amplified in the presence of environmental heat, with studies reporting increased rates of psychiatric emergency department visits among adolescents during periods of elevated temperature, a life stage characterized by rapid neurodevelopment and hormonal changes [10], [11]. Heat stress is also known to disrupt sleep, impair emotion regulation and concentration, processes that are essential to mood regulation and psychological well-being for young people [13], [14].

Beyond adolescence, emerging evidence suggests that heat exposure is associated with a broader range of adverse mental health outcomes among females across the life course, although findings remain heterogeneous and are derived from diverse study designs and outcome measures. Longitudinal studies in China and the United States have reported associations between elevated temperatures and depressive symptoms, with some evidence of strong effects among females and postpartum depressive symptoms among low-income Hispanic/Latina women during pregnancy [39], [40]. Studies from the Netherlands and Spain similarly suggest associations between heat exposure and anxiety-related symptoms, psychiatric morbidity, and internalizing problems, while investigations in France, Canada, and Australia have reported relationships between heat wave exposure and emergency department visits for psychotic disorders and schizophrenia admissions [41], [42], [43], [44], [45]. While these findings suggest that heat may adversely affect multiple dimensions of mental health, little research has examined these relationships during adolescence or young adulthood, and none have examined menstrual health as a potential modifier of heat-related mental health outcomes during these sensitive developmental stages.

One plausible, yet unexplored pathway through which heat may influence mental health during adolescence and young adulthood is menstrual physiology. For menstruating adolescents, hormonal fluctuations may modify responses to heat exposure through effects on thermoregulation, pain sensitivity, sleep quality, and emotional processing [23], [33]. Heat exposure during the luteal phase, when body temperature is naturally elevated, may intensify fatigue, irritability, and mood disturbances [34]. Although evidence supports independent associations between heat exposure, menstrual physiology, and mental health, their combined effects have not been empirically examined.

### 3.3 Climate change and environmental heat: the environmental driver

The need to understand these relationships is becoming increasingly urgent as climate change shifts population-level heat exposure towards conditions that adolescents and young adults will experience more frequently throughout their lives. Extreme heat is increasing in duration, frequency and intensity worldwide with climate change [44]. Evidence also suggests that often the size of extreme heat events is increasing in geographical extent and therefore a greater proportion of a population is exposed to sustained periods of heat stress [8], [45]. Extreme heat may exacerbate existing health and social disparities by disproportionately affecting populations with limited physiological or social capacity to adapt [46], [47]. Although the elderly, infants, and those with pre-existing chronic conditions, are widely recognized as being at increased risk, adolescence and young adulthood represent comparatively overlooked life stages despite substantial physiological, hormonal, and psychosocial changes that may influence responses to heat [32].

Understanding heat-related risks among adolescent girls and young women is further complicated by longstanding gaps in heat physiology research. Historically, physiological studies of heat exposure have been disproportionately carried out on males than females, limiting understanding of how hormonal transitions, menstrual physiology, and reproductive life stages influence thermoregulation [46]. For example, the Universal Thermal Climate Index (UTCI), one of the most widely used heat stress metrics, is based upon a body model of an average European man [48]. As a result, commonly used heat warning thresholds may not adequately reflect physiological responses among adolescent girls and young women.

An emerging body of work has begun to recognize menstrual health as an important but underexamined dimension of climate resilience, highlighting the biological, psychosocial, and structural pathways through which climate change may affect menstrual health and calling for more integrated and equitable adaptation strategies [49]. However, these emerging frameworks have not yet explicitly considered how environmental heat, menstrual health, and mental health intersect during adolescence and young adulthood. Addressing this gap requires an integrated life-course perspective that considers these pathways together rather than in isolation.

### 3.4 Linking biological, social, and environmental pathways: a conceptual framework

**Fig. 1:**
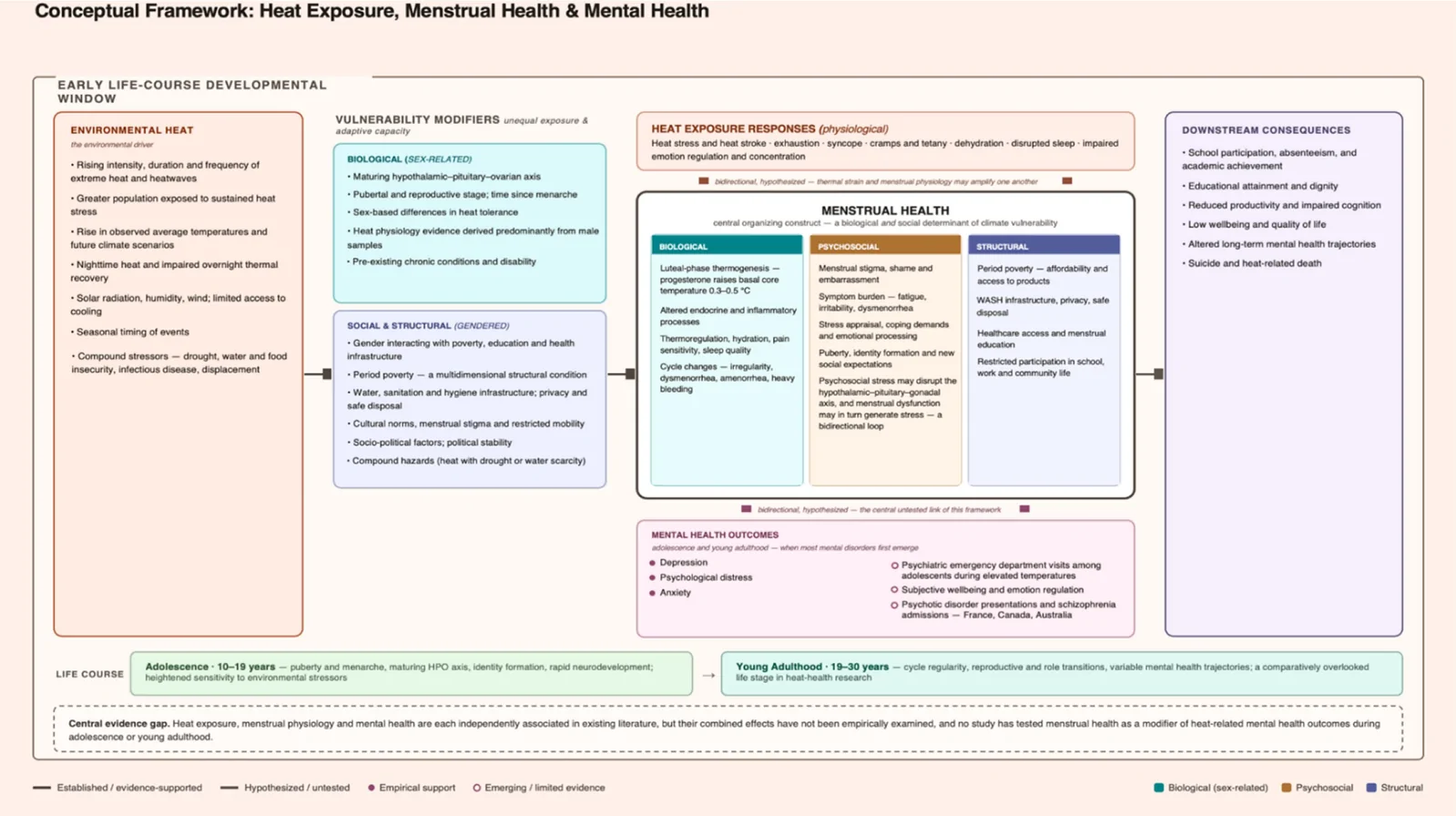
Conceptual framework of heat exposure, menstrual health and mental health and the different factors that influence health outcomes and consequences drawn together from the narrative critical review. It covers environmental heat as the exposure, biological and social and structural vulnerabilities and different biological psychosocial and structural aspects of menstrual health.

Building on the preceding evidence, we propose an integrated conceptual framework that organizes the biological, psychosocial, and structural pathways through which environmental heat may influence menstrual health and, in turn, adolescent and young adult mental health (Fig. 1). Rather than acting through a single mechanism, heat exposure is likely to interact with hormonal physiology, psychosocial experiences, and broader environmental and structural conditions across adolescence and young adulthood.

From a biological perspective, the hypothalamus serves as a central regulator of both thermoregulation and reproductive hormones. Heat stress, psychosocial stress, and menstrual cycle fluctuations each activate the hypothalamic–pituitary–adrenal (HPA) axis, altering cortisol release with downstream effects on mood and physiology [50], [51]. Inflammatory signaling may provide another shared pathway. Proinflammatory cytokines, such as interleukin-6 (IL-6) and tumor necrosis factor-α (TNF-α), are elevated in both depression and heat stroke, suggesting biologically plausible immunological mechanisms linking these conditions [52], [53].

Emerging evidence also points to the gut–brain axis as a mediator. Sex hormones influence gut microbiota composition, and menstrual irregularities have been linked to microbial dysbiosis, which is itself associated with anxiety and depressive symptoms [54], [55]. Environmental stressors, such as extreme heat events, may exacerbate these disruptions, amplifying effects on both mood and menstrual health.

#### 3.4.1 Psycho-social pathways

Psychosocial pathways may further shape how heat and menstruation are experienced during adolescence and young adulthood. Menstrual pain and discomfort during periods of extreme heat may be compounded by menstrual stigma, shame, and concerns about managing menstruation discreetly, particularly in settings with limited privacy or inadequate menstrual resources. These experiences may restrict participation in school, work, physical activity, and social engagement, contributing to absenteeism, social isolation, and heightened psychological stress. Heat-related sleep disruption may further exacerbate emotional dysregulation, anxiety, and depressive symptoms, particularly during this sensitive developmental period. These experiences are embedded within broader structural conditions, including period poverty and unequal access to water, sanitation, and menstrual resources, that influence both exposure to heat and the capacity to manage menstruation safely and with dignity [26], [28].

#### 3.4.2 Structural pathways

Structural conditions influence both exposure and adaptive capacity. Period poverty, inadequate water, sanitation and hygiene (WASH) infrastructure, limited access to cooling, housing quality, school environments, health-care access, and broader socioeconomic inequities may influence an individual's ability to manage menstruation safely and comfortably during extreme heat. These structural factors may therefore modify both menstrual health and mental health outcomes.

Importantly, these pathways are unlikely to operate independently. Biological responses to heat may be modified by psychosocial experiences such as stigma and stress, while structural conditions including period poverty, access to cooling, housing quality, and water and sanitation infrastructure shape both heat exposure and the capacity to manage menstruation safely and with dignity. These interacting pathways may cumulatively influence mental health during adolescence and young adulthood. By situating these interrelated pathways within a life-course perspective, the framework identifies adolescence and young adulthood as periods of heightened susceptibility while providing a foundation for generating testable hypotheses regarding the effects of environmental heat on menstrual health and mental wellbeing (Fig. 1).

### 3.5 Towards a testable framework: research priorities and pathways

**Fig. 2:**
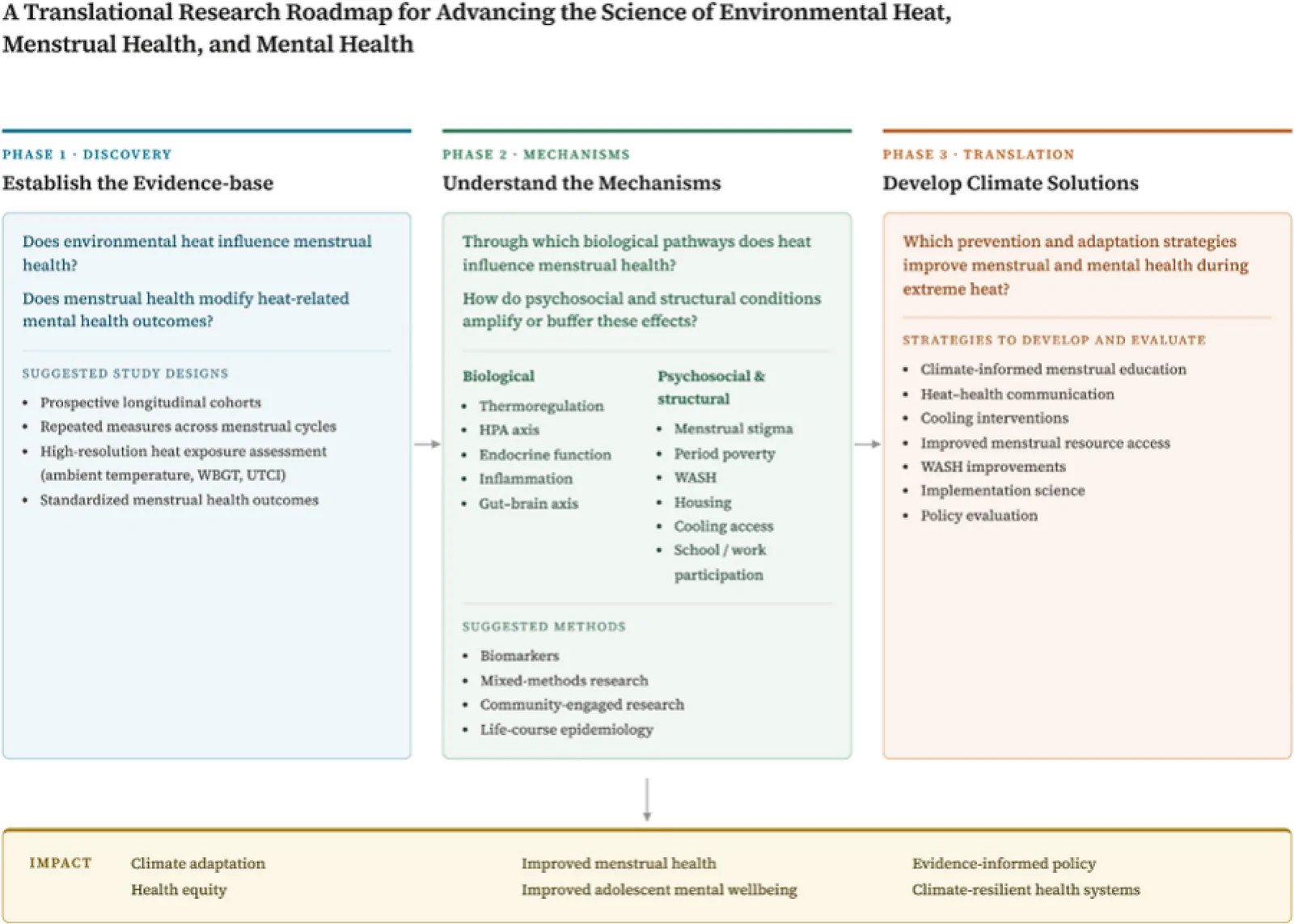
Is a roadmap of different phases to explore the evidence linking menstrual and mental health with heat exposure and moving this forward into creating actionable climate solutions and the positive impact this can have.

The conceptual framework presented in Fig. 1 identifies several biologically plausible and socially mediated pathways linking environmental heat, menstrual health, and mental health. To the best of our knowledge there is no empirical evidence linking extreme heat, menstruation, and adolescent and young adult mental health. To start providing evidence towards these critical gaps, we propose a gender-responsive research agenda that recognizes menstruation as a potential climate-sensitive health issue and adolescent and young adult mental health and wellbeing as central to climate adaptation and health equity. Fig. 2 provides a conceptual roadmap for operationalizing these gaps within future epidemiologic, clinical, and implementation studies. Fig. 2 translates the proposed conceptual framework into a research roadmap, outlining priority research questions alongside recommended study designs, methodological approaches, and intervention strategies to advance the field.

## 4 Conclusion

Extreme heat is a growing global health threat, yet its intersection with menstruation and mental health in adolescence and young adulthood remains critically overlooked. The biological, psychological, and structural dimensions of menstrual health have received limited attention in climate and health research, despite their potential to shape vulnerability during a sensitive developmental period. For adolescent girls and young women, who already face heightened risk of depression, anxiety, and psychosocial stressors, the added burden of heat exposure represents a neglected but urgent area of research inquiry. Our critical narrative review highlights biologically plausible and socially mediated pathways through which environmental heat may influence menstrual health and, in turn, mental health, while identifying priorities for future research and intervention. Advancing this research agenda has important implications for achieving Sustainable Development Goal 3 (Good Health and Well-being) by improving prevention strategies and strengthening climate-resilient health systems for adolescents and young adults. Because menstrual health is both a biological process and a gendered social experience, this agenda may also contribute to SDG5 (Gender Equality) and SDG13 (Climate Action) by informing adaptation strategies for populations disproportionately affected by extreme heat.

## CRediT authorship contribution statement

**C. Brimicombe:** Writing – review & editing, Writing – original draft, Validation, Resources, Project administration, Methodology, Investigation, Conceptualization. **J.D. Runkle:** Writing – review & editing, Writing – original draft, Validation, Supervision, Methodology, Conceptualization. **B. Mouchtouri:** Writing – review & editing. **C. Cattle:** Writing – review & editing, Writing – original draft, Resources, Conceptualization. **C. Part:** Writing – review & editing, Resources. **F. Kalala:** Writing – review & editing, Resources. **H. Marshall:** Writing – review & editing, Resources. **I. Voulgaridi:** Writing – review & editing, Resources. **K. Wan:** Writing – review & editing, Resources. **M. Tembo:** Writing – review & editing, Resources. **S. Hajat:** Writing – review & editing, Resources. **V. Simms:** Writing – review & editing, Resources. **P. Banati:** Writing – review & editing, Validation, Supervision, Resources, Methodology, Conceptualization.

## Declaration of competing interest

There is no declaration of interests to disclose.

## References

[1] Kessler R.C., Amminger G.P., Aguilar-Gaxiola S., Alonso J., Lee S., Üstün T.B. (2007). Age of onset of mental disorders: a review of recent literature. Curr Opin Psychiatry.

[2] Jones P.B. (2013). Adult mental health disorders and their age at onset. Br J Psychiatry.

[3] Stumper A., Alloy L.B. (2021). Associations between pubertal stage and depression: a systematic review of the literature. Child Psychiatry Hum Dev.

[4] Thapar A., Eyre O., Patel V., Brent D. (2022). Depression in young people. Lancet.

[5] Patton G.C., Sawyer S.M., Santelli J.S., Ross D.A., Afifi R., Allen N.B. (2016). Our future: a lancet commission on adolescent health and wellbeing. Lancet.

[6] Lee F.S., Heimer H., Giedd J.N., Lein E.S., Šestan N., Weinberger D.R. (2014). Adolescent mental health—opportunity and obligation. Science (1979).

[7] Mora C., Dousset B., Caldwell I.R., Powell F.E., Geronimo R.C., Bielecki C.R. (2017). Global risk of deadly heat. Nat Clim Chang.

[8] Perkins-Kirkpatrick S.E., Lewis S.C. (2020). Increasing trends in regional heatwaves. Nat Commun.

[9] Cramer M.N., Gagnon D., Laitano O., Crandall C.G. (2022). Human temperature regulation under heat stress in health, disease, and injury. Physiol Rev.

[10] Nori-Sarma A., Sun S., Sun Y., Spangler K.R., Oblath R., Galea S. (2022). Association between ambient heat and risk of emergency department visits for mental health among US adults, 2010 to 2019. JAMA Psychiatry.

[11] Essers E., Kusters M., Granés L., Ballester J., Petricola S., Lertxundi N. (2025). Temperature exposure and psychiatric symptoms in adolescents from 2 European birth cohorts. JAMA Netw Open.

[12] Rony M.K.K., Alamgir H.M. (2023). High temperatures on mental health: recognizing the association and the need for proactive strategies—a perspective. Health Sci Rep.

[13] Minor K., Bjerre-Nielsen A., Jonasdottir S.S., Lehmann S., Obradovich N. (2022). Rising temperatures erode human sleep globally. One Earth.

[14] Taylor L., Watkins S.L., Marshall H., Dascombe B.J., Foster J. (2016). The impact of different environmental conditions on cognitive function: a focused review. Front Physiol.

[15] Cosh S.M., Ryan R., Fallander K., Robinson K., Tognela J., Tully P.J. (2024). The relationship between climate change and mental health: a systematic review of the association between eco-anxiety, psychological distress, and symptoms of major affective disorders. BMC Psychiatry.

[16] Runkle J.D., Reed C., Weidner K., Rothschild J., Chandrasekhar T., Sugg M.M. (2025). Assessing the impact of heatwaves on emergency visits for major depression and suicidal ideation in youth with attention-deficit/hyperactivity disorder. PLOS Mental Health.

[17] Runkle Jennifer D., Herbst Kelsey, Ryan Sophie, Sewell Kelly, Mallare Ashley, Berry Ian (2025). Eco-anxiety, climate concern, and fatalistic outlooks: Insights from US crisis text conversations on climate distress. J Clim Chang Health.

[18] Baker F.C., Siboza F., Fuller A. (2020). Temperature regulation in women: effects of the menstrual cycle. Temperature.

[19] Mirin A.A. (2021). Gender disparity in the funding of diseases by the U.S. National Institutes of Health. J Women’s Health.

[20] Barnett-Page E., Thomas J. (2009). Methods for the synthesis of qualitative research: a critical review. BMC Med Res Methodol.

[21] Vergunst F., Berry H.L. (2022). Climate change and children’s mental health: a developmental perspective. Clin Psychol Sci.

[22] Tatsumi T., Sampei M., Saito K., Honda Y., Okazaki Y., Arata N. (2020). Age-dependent and seasonal changes in menstrual cycle length and body temperature based on big data. Obstet Gynecol.

[23] Marsh S.A., Jenkins D.G. (2002). Physiological responses to the menstrual cycle. Sports Med.

[24] Gilworth R.E., Skinner B.D., Hodgkiss D.D., Lucas S.J.E., Lucas R.A.I. (2025). Mapping the evidence on the impact of heat stress on exercise and work performance in females: a scoping review. Front Physiol.

[25] Hannan F.M., Leow M.K.S., Lee J.K.W., Kovats S., Elajnaf T., Kennedy S.H. (2024). Endocrine effects of heat exposure and relevance to climate change. Nat Rev Endocrinol.

[26] Khofi L. (2024). Period poverty is a continuing global challenge. Nat Hum Behav.

[27] Sommer M., Hirsch J.S., Nathanson C., Parker R.G. (2015). Comfortably, safely, and without shame: defining menstrual hygiene management as a public health issue. Am J Public Health.

[28] Zaman M.N.U., Takashima R., Sai A., Yamauchi T. (2025). Period poverty: menstrual information, product selection, and disposal among urban female nursing students in Bangladesh. Health Place.

[29] Bwalya B.B., Mwansa A., Amanzi P., Ngongola C., Meki-Kombe C. (2025). A comparative study of menstrual poverty among urban and rural female learners in government schools of Zambia. BMC Womens Health.

[30] Vågenes V., Grevstad C. (2025). Menstruation and period poverty as an obstacle for girls’ equal participation in education, Tanzania. BMC Public Health.

[31] IRISE International (2025).

[32] Atkinson H.G., Bruce J. (2015). Adolescent girls, human rights and the expanding climate emergency. Ann Glob Health.

[33] Rothschild J., Haase E. (2023). Women’s mental health and climate change part II: socioeconomic stresses of climate change and eco-anxiety for women and their children. Int J Gynecol Obstet.

[34] Majeed H., Lee J. (2017). The impact of climate change on youth depression and mental health. Lancet Planet Health.

[35] Kollipaka R., Arounassalame B., Lakshminarayanan S., Lakshminarayanan &.S. (2013). Does psychosocial stress influence menstrual abnormalities in medical students?. J Obstet Gynaecol (Lahore).

[36] Chao M., Menon C., Elgendi M. (2022). Menstrual cycles during COVID-19 lockdowns: a systematic review and meta-analysis. Front Reprod Health.

[37] Nagma S., Kapoor G., Bharti R., Batra A., Batra A., Aggarwal A. (2015). To evaluate the effect of perceived stress on menstrual function. J Clin Diagn Res.

[38] Loucks A.B., Redman L.M. (2004). The effect of stress on menstrual function. Trends Endocrinol Metab.

[39] Jahan S., Wraith D. (2021). Immediate and delayed effects of climatic factors on hospital admissions for schizophrenia in Queensland Australia: a time series analysis. Environ Res.

[40] Hu Y., Pardo N., Yang X., Yang T., Xu Y., Khalili R. (2025). Prenatal exposure to heat stress and postpartum depressive symptoms in the MADRES cohort in urban Los Angeles. Environ Res.

[41] Christodoulou N., Laaidi K., Fifre G., Lejoyeux M., Ambar Akkaoui M., Geoffroy P.A. (2024). Heatwaves and mental disorders: a study on national emergency and weather services data. Eur J Psychiatry.

[42] Tupinier Martin F., Boudreault J., Campagna C., Lavigne É., Gamache P., Tandonnet M. (2024). The relationship between hot temperatures and hospital admissions for psychosis in adults diagnosed with schizophrenia: a case-crossover study in Quebec, Canada. Environ Res.

[43] Essers E., Kusters M., Granés L. (2025). Temperature exposure and psychiatric symptoms in adolescents from 2 European birth cohorts. JAMA Netw Open.

[44] IPCC, Masson-Delmotte V., Zhai P., Pirani A., Connors S.L., Péan C. (2021). https://www.ipcc.ch/.

[45] Thompson V., Mitchell D., Hegerl G.C., Collins M., Leach N.J., Slingo J.M. (2023). The most at-risk regions in the world for high-impact heatwaves. Nat Commun.

[46] Brimicombe C., Gao C., Otto I.M. (2024). Vulnerable to heat stress: gaps in international standard metric thresholds. Int J Biometeorol.

[47] McGregor G.R., Vanos J.K. (2018). Heat: a primer for public health researchers. Public Health.

[48] Jendritzky G., de Dear R., Havenith G. (2012). UTCI-why another thermal index?. Int J Biometeorol.

[49] Muralidharan A., Broekhuijsen M., Lisondra Lady, Guru A., Haver J., Irfan S. (2025). The ripple effect: impacts of climate change on menstrual health and paths to resilience. Front glob Womens Health.

[50] McEwen B.S. (2015). Biomarkers for assessing population and individual health and disease related to stress and adaptation. Metabolism.

[51] Allen R.E., Rogozinska E., Cleverly K., Aquilina J., Thangaratinam S. (2014). Abnormal blood biomarkers in early pregnancy are associated with preeclampsia: a meta-analysis. Eur J Obstet Gynecol Reprod Biol.

[52] Wang C.C., Tsai M.K., Chen I.H., Hsueh C.W., Shiang J.C. (2008). Heat stroke. J Int Med Taiwan.

[53] Dantzer R., O’Connor J.C., Freund G.G., Johnson R.W., Kelley K.W. (2007). From inflammation to sickness and depression: when the immune system subjugates the brain. Nat Rev Neurosci.

[54] Koebele S.V., Bimonte-Nelson H.A. (2017).

[55] Sherwin E., Bordenstein S.R., Quinn J.L., Dinan T.G., Cryan J.F. (2019). Microbiota and the social brain. Science (1979).

